# Role of the *miR-17∼92* cluster family in cerebellar and medulloblastoma development

**DOI:** 10.1242/bio.20146734

**Published:** 2014-06-13

**Authors:** Frederique Zindy, Daisuke Kawauchi, Youngsoo Lee, Olivier Ayrault, Leila Ben Merzoug, Peter J. McKinnon, Andrea Ventura, Martine F. Roussel

**Affiliations:** 1 Department of Tumor Cell Biology, Danny Thomas Research Center, St Jude Children’s Research Hospital, Memphis, TN 38105-3678, USA; 2 Department of Genetics, Danny Thomas Research Center, St Jude Children’s Research Hospital, Memphis, TN 38105-3678, USA; 3 Cancer Biology and Genetics Program, Memorial Sloan Kettering Cancer Center, New York, NY 10065, USA; * Present address: Division of Pediatric Neurooncology, German Cancer Research Center (DKFZ), Im Neuenheimer Feld 580, 69120 Heidelberg, Germany.; ‡ Present address: Genomic Instability Research Center, Ajou University, School of Medicine, Suwon 443-749, South Korea.; § Present address: Institut Curie/CNRS UMR 3306/INSERM U1005 – Building 110 – Centre Universitaire, 91405 Orsay, Cedex, France.

**Keywords:** MicroRNA, *miR-17∼92* and *miR-106b∼25* clusters, Cerebellum, Development, Nestin, Medulloblastoma, Granule neuron progenitors (GNPs)

## Abstract

The *miR-17∼92* cluster family is composed of three members encoding microRNAs that share seed sequences. To assess their role in cerebellar and medulloblastoma (MB) development, we deleted the *miR-17∼92* cluster family in Nestin-positive neural progenitors and in mice heterozygous for the Sonic Hedgehog (SHH) receptor Patched 1 (*Ptch1^+/−^*). We show that mice in which we conditionally deleted the *miR-17∼92* cluster (*miR-17∼92^floxed/floxed^*; *Nestin-Cre^+^*) alone or together with the complete loss of the *miR-106b∼25* cluster (*miR-106b∼25^−/−^*) were born alive but with small brains and reduced cerebellar foliation. Remarkably, deletion of the *miR-17∼92* cluster abolished the development of SHH-MB in *Ptch1^+/−^* mice. Using an orthotopic transplant approach, we showed that granule neuron precursors (GNPs) purified from the cerebella of postnatal day 7 (P7) *Ptch1^+/−^*; *miR-106b∼25^−/−^* mice and overexpressing *Mycn* induced MBs in the cortices of naïve recipient mice. In contrast, GNPs purified from the cerebella of P7 *Ptch1^+/−^*; *miR-17∼92^floxed/floxed^*; *Nestin-Cre^+^* animals and overexpressing *Mycn* failed to induce tumors in recipient animals. Taken together, our findings demonstrate that the *miR-17∼92* cluster is dispensable for cerebellar development, but required for SHH-MB development.

## INTRODUCTION

The cerebellum develops in the mouse from embryonic day 9 (E9) with the cerebellar anlage forming from the roof (the alar plates) of the metencephalon. It is composed of different types of neurons that arise from the ventricular zone (VZ) of the cerebellar neuroepithelium localized on the roof of the fourth ventricle, and from the rostral rhombic lip (rRL), localized at the posterior edge of the cerebellar anlage (Hatten and Roussel, 2011). In the mouse, granule neuron progenitors (GNPs) are born in the rRL (E11–E16) and migrate along the surface of the developing cerebellum over the Purkinje cells, which are born in the VZ (E11–E13), to form the external granule layer (EGL) (E13–E16). After birth, Sonic Hedgehog (SHH), secreted by the Purkinje cells, promotes the proliferation of GNPs, which peaks between postnatal days 5 (P5) and P7. Subsequently, GNPs exit the cell cycle, migrate inwardly through the Purkinje cell layer and settle as postmitotic neurons in the internal granular layer (IGL). By 3 weeks, the mouse cerebellum is fully formed consisting of ten folia separated by fissures (Hatten and Roussel, 2011). Constitutive activation of the SHH signaling pathway leads to defects in cell cycle exit, migration and differentiation, which, in turn, induce medulloblastoma (MB). This SHH-subgroup of MBs (SHH-MB) represents ∼25% of all human cases (Taylor et al., 2012).

MicroRNAs (miRNAs) are ∼22 nucleotides long non-coding RNAs. They are derived from pri-miRNAs that are processed by Drosha and DGCR8 into pre-miRNAs in the nucleus, and then translocated into the cytoplasm where they are converted into mature miRNAs by the processing enzyme Dicer. In turn, single-stranded miRNAs are loaded into the RNA-induced silencing complex (RISC) to bind the 3′-untranslated region (3′-UTR) of mRNAs to inhibit their translation or degradation (Carmell and Hannon, 2004; Kim, 2005).

**Fig. 1.**
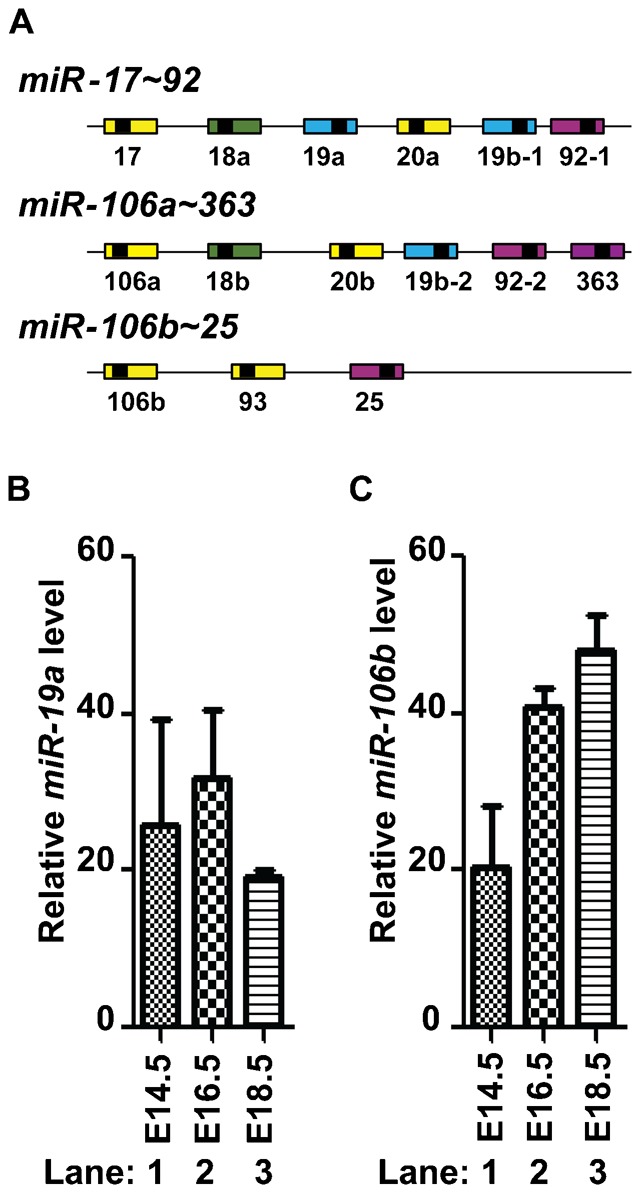
Expression of *miR-19a* and *miR-106b* encoded by the *miR-17∼92* cluster family in cerebella during embryogenesis. (A) Schematic representation of the three microRNA clusters. *MiR-17∼92*, *miR-106a∼363* and *miR-106b∼25* clusters encode 6, 6 and 3 mature microRNAs (colored boxes), respectively. MicroRNAs sharing the same seed sequence are represented by boxes of the same color. Relative levels of mature microRNAs *miR-19a* (B) and *miR-106b* (C) were determined by Q-RT-PCR on total RNA extracted from total cerebella of wild-type mice (lanes 1–3) at E14.5 (lanes 1), E16.5 (lanes 2) and E18.5 (lanes 3).

Abnormal expression of miRNAs is often seen in cancers. MicroRNAs encoded by the *miR-17∼92* cluster, also called *oncomiR-1*, are overexpressed in various cancers (Concepcion et al., 2012; Mogilyansky and Rigoutsos, 2013) including mouse and human medulloblastomas with constitutively activated SHH signaling (Uziel et al., 2009; Northcott et al., 2009). The *miR-17∼92* cluster encoded by chromosome 14 in the mouse (13 in humans) has two paralogs, the *miR-106b∼25* and *miR-106a∼363* clusters, each of which is located on different chromosomes (Fig. 1A). The *miR-106b∼25* cluster is encoded on chromosome 5 in the mouse (7 in humans) while the *miR-106a∼363* cluster maps to chromosome X in mice and humans (Concepion et al., 2012; Mogilyansky and Rigoutsos, 2013). Mice lacking the *miR-17∼92* cluster die shortly after birth from lung and heart defects while mice lacking each of its two paralogs do not show any obvious phenotypes (Ventura et al., 2008). However, the *miR-17∼92* and *miR-106b∼25* clusters share overlapping functions since mice with combined deletion exhibit a more profound phenotype than those lacking *miR-17∼92* alone (Ventura et al., 2008). The *miR-17∼92* cluster is a downstream target of Myc (c-Myc) (O'Donnell et al., 2005) and Mycn (Northcott et al., 2009; de Pontual et al., 2011). Mice lacking one copy of *miR-17∼92* show skeletal and growth defects recapitulating the Feingold syndrome observed in patients harboring *MYCN* mutations or hemizygous deletion of *MIR-17∼92* (de Pontual et al., 2011). The *miR-17∼92* cluster is expressed in proliferative GNPs but not in post-mitotic granule neurons. Overexpression of the *miR-17∼92* cluster in GNPs heterozygous for the SHH receptor, *Patched 1* (*Ptch1^+/−^*), induces early onset of SHH-MB formation after orthotopic transplant in the cortices of naïve recipient animals (Uziel et al., 2009). Similarly when overexpressed in wild-type GNPs, the *miR-17∼92* cluster collaborates with SHH signaling to provide GNPs with a proliferative advantage (Northcott et al., 2009). These results suggested that, besides its role in SHH-MBs, the *miR-17∼92* cluster might play a role in cerebellar development. We here show that the *miR-17∼92* cluster and its paralog the *miR-106b∼25* cluster, are differently required for cerebellar and medulloblastoma development.

## MATERIALS AND METHODS

### Mice

Mouse lines carrying conditional alleles of *miR-17∼92* (*miR-17∼92^floxed/floxed^*) (Ventura et al., 2008), or lacking *miR-106b∼25* (*miR-106b∼25^−/−^*) (Ventura et al., 2008) were generously provided by Dr Tyler Jacks (Boston, MA, USA). The transgenic line in which the Cre recombinase is expressed under the promoter of the rat *Nestin* gene (*Nestin-Cre*) (stock number 003771) (Tronche et al., 1999) and C57BL/6 mice were obtained from the Jackson Laboratory (Bar Harbor, ME, USA). *Ptch1^+/−^* mice in a *Cdkn2c*-null background (*Ptch1^+/−^*; *Cdkn2c^−/−^*) were previously described (Uziel et al., 2005). All mice were maintained on a mixed 129×C57BL/6 background. CD-1 *nu/nu* mice were obtained from Charles River (Wilmington, MA, USA). Mice were housed in an accredited facility of the Association for Assessment and Accreditation of Laboratory Animal Care International (AAALAC) in accordance with National Institute of Health guidelines. The Animal Care and Use Committee (ACUC) of St Jude Children's Research Hospital approved all procedures.

### Histology, immunohistochemistry, proliferation assays and X-gal staining

Heads of embryos or whole brains from mice were harvested, fixed overnight in 4% paraformaldehyde (PFA) in phosphate buffered saline (PBS) at 4°C, soaked in 30% sucrose in PBS until the tissues sank to the bottom of the tube and embedded in Optimal Cutting Temperature (OCT) compound. Frozen 12 µm sections were collected on Fisherbrand superfrost plus slides using a cryostat. Slides were stained with Hematoxylin and Eosin (H&E) or antibodies, as previously described (Uziel et al., 2005). The following antibodies used were: 5-Bromo-2-Deoxy-Uridine (BrdU) (SC-32323; Santa Cruz, Dallas, TX, USA; 1/1000 dilution), p27^Kip1^ (610242; BD Biosciences, San Jose, CA; 1/200 dilution), cyclin D2 (SC-593; Santa Cruz; 1/50 dilution), NeuN (MAB377; EMD Millipore, Billerica, MA, USA; 1/200 dilution), Ki67 (NLC-Ki67p; Leica microsystems, Buffalo Grove, IL; 1/1000 dilution) and GABA(A) receptor α6 subunit (AB5610; EMD Millipore; 1/200 dilution). BrdU (100 mg/kg) was injected intra-peritoneally two hours before harvesting the brain. For X-gal staining, whole brains from 3-week-old animals were fixed in 2% PFA in PBS at 4°C for 3 hours. Fixed brains were washed twice in PBS and then incubated in the “Rinse” buffer (100 mM sodium phosphate pH 7.3, 2 mM MgCl_2_, 0.01% sodium deoxycholate, 0.02% IGEPAL CA-360) for 5 minutes at room temperature. Brains were stained with X-gal staining solution (“Rinse” buffer containing 5 mM potassium ferricyanide, 5 mM potassium ferrocyanide, 1 mg/ml X-galactoside 5-bromo-4-chloro-3-indolyl-beta-D-galactopyranoside (X-gal) (Invitrogen, Life technologies, Grand Island, NY, USA), at 37°C for 4 hours.

### Quantitative-reverse transcriptase-polymerase chain reaction (Q-RT-PCR) and Affymetrix microarrays analysis

Purification of GNPs from P7 cerebella, RNA extraction from dissected embryonic and postnatal (P4 and P7) cerebella or from purified GNPs, and Q-RT-PCR for *miR-19a* and *miR-106b* were performed, as previously described (Uziel et al., 2005; Uziel et al., 2009). For comparative gene expression analysis, RNAs from cerebella of P4 and P7 mice were subjected to hybridization using Affymetrix Mouse GeneChip MG430PM (Affymetrix, Santa Clara, CA). Principal component analysis (PCA) and gene set enrichment analysis (GSEA) were performed using Partek Genomics Suite 6.6 software (St Louis, MO) and GSEA software (Broad Institute, Cambridge, MA), respectively.

### Orthotopic transplantation

GNPs were purified from P7 cerebella and infected with retroviruses encoding Mycn and the red fluorescent protein (RFP), as previously described (Kawauchi et al., 2012). 48 hours after infection, 2×10^6^ infected GNPs were injected into the cortices of naïve recipient CD-1 *nu/nu* mice. The rate of infection was assessed by fluorescence activated cell sorting (FACS). The percentage of RFP positive (RFP+) cells ranged from 30 to 50%.

### Statistics

Statistical significance was determined using GraphPad Prism software (version 5.0). Data were shown as mean ± s.e.m. P-values <0.05 were used as significance threshold from unpaired two-tailed Student's *t* test. For the survival curves, p-values were determined with a log-rank (Mantel Cox) test.

## RESULTS

### Co-inactivation of the *miR-17∼92* and *miR-106b∼25* clusters reduced cerebellar size and foliation by limiting proliferation

We previously found that microRNAs encoded by the *miR-17∼92* and *miR-106b∼25*, but not the *miR-106a∼363*, clusters are expressed in proliferating GNPs in the postnatal cerebellum (Uziel et al., 2009). Because these miRNAs were expressed in developing cerebella from E14.5 to birth (Fig. 1B,C, lanes 1–3), we assessed their role during cerebellar development. Since *miR-17∼92*-null mice die shortly after birth (Ventura et al., 2008), the *miR-17∼92* cluster was conditionally deleted in Nestin-positive neural progenitors using a *Nestin-Cre* transgenic mouse (*miR-17∼92^floxed/floxed^*; *Nestin-Cre^+^*) in a wild-type *miR-106b∼25* (referred as *miR-17∼92*cKO) or in a *miR-106b∼25*-null (referred as *miR-17∼92*cKO; *miR-106b∼25*KO) background. We also generated *miR-17∼92^floxed/floxed^*; *Nestin-Cre^−^*; *miR-106b∼25^−/−^* (referred as *miR-106b∼25*KO) and *miR-17∼92^floxed/floxed^*; *Nestin-Cre^−^*; *miR-106b∼25^+/+^* (referred as control) littermates. Mice of all genotypes were born at a Mendelian ratio.

**Fig. 2.**
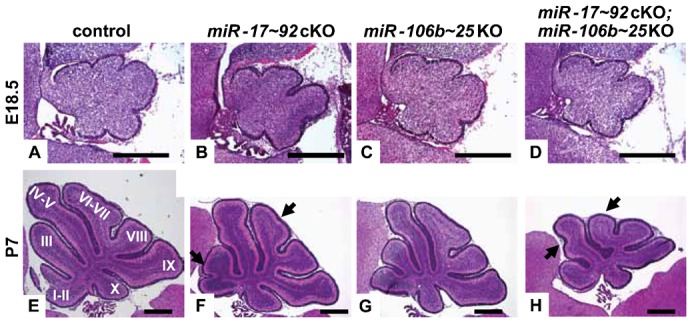
Progressive foliation defects in cerebella of mice lacking the *miR-17∼92* and *miR-106b∼25* clusters. Mid-sagittal sections through the cerebellum of a control (A,E), *miR-17∼92*cKO (B,F), *miR-106b∼25*KO (C,G) and *miR-17∼92*cKO; *miR-106b∼25*KO (D,H) mouse at E18.5 (A–D) and P7 (E–H) of age were stained by H&E. The ten vermis lobules are indicated by Roman numbers. Arrows indicate the area of abnormal foliation. Scale bars: 500 µm.

At E18.5, cerebella of all 4 genotypes were indistinguishable displaying the 5 cardinal lobes separated by four principal fissures (Fig. 2A–D) despite complete Cre-mediated recombination of the two *miR-17∼92* alleles (supplementary material Fig. S1A) and reduced levels of *miR-19a* in cerebella of *miR-17∼92*cKO mice (supplementary material Fig. S1B, lane 2).

At P7, the ten folia were observed in the cerebella of control mice (Fig. 2E). However, cerebella of *miR-17∼92*cKO; *miR-106b∼25*KO mice and, to a lesser extent, cerebella of *miR-17∼92*cKO mice, demonstrated foliation defects mainly in folia VI–VII and folia I–V (compare Fig. 2H and Fig. 2F with Fig. 2E, respectively, see arrows), while the folia in the cerebella of *miR-106b∼25*KO mice appeared similar to those of control mice (compare Fig. 2G with Fig. 2E).

**Fig. 3.**
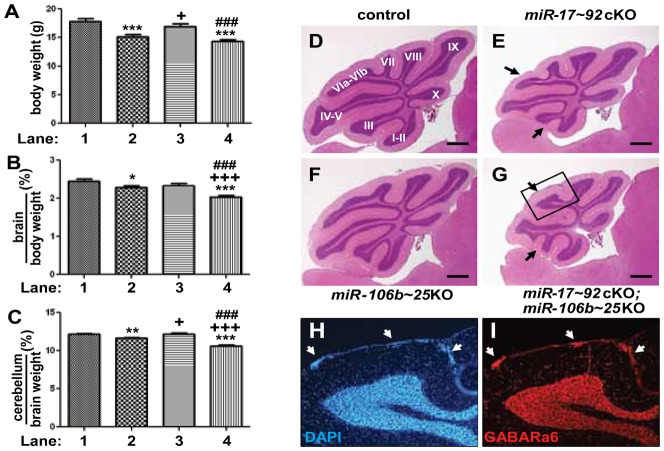
Mice lacking the *miR-17∼92* and *miR-106b∼25* clusters have small brains and small cerebella. (A) Body weight, (B) ratio (expressed in %) of brain versus body weight and (C) ratio (expressed in %) of cerebellum versus brain weight of control (lanes 1; n = 23), *miR-17∼92*cKO (lanes 2; n = 45), *miR-106b∼25*KO (lanes 3; n = 11) and *miR-17∼92*cKO; *miR-106b∼25*KO (lanes 4; n = 31) mice at 1 month of age. (* and +) p-values <0.05, (**) p-values <0.01, (***, +++ and ###) p-values <0.001. P-values were calculated by comparing lanes 2–4 to lane 1 (*), lanes 3 and 4 to lane 2 (+) and lane 4 to lane 3 (#). Mid-sagittal sections through the cerebellum of control (D), *miR-17∼92*cKO (E), *miR-106b∼25*KO (F) and *miR-17∼92*cKO; *miR-106b∼25*KO (G) mouse at 1 month of age stained by H&E. The ten vermis lobules are indicated by Roman numbers. Arrows indicate foliation abnormalities. Enlarged view of boxed area in panel G is presented in panels H and I. Clusters of mature granule neurons (white arrows) on the surface of the molecular layer were stained with DAPI (H) and an antibody against GABA(A) receptor α6 subunit (GABARa6) (I). Scale bars: 500 µm.

At one month of age, *miR-17∼92*cKO and *miR-17∼92*cKO; *miR-106b∼25*KO mice showed statistically smaller body weight (Fig. 3A, lanes 2 and 4 versus lane 1, respectively), brains and cerebella (Fig. 3B,C, lanes 2 and 4 versus lane 1, respectively) compared to control mice. In contrast, the size of *miR-106b∼25*KO mice was similar to control animals (Fig. 3A–C, lane 3 versus lane 1). This demonstrated that, despite the small brain size of the *miR-17∼92*cKO; *miR-106b∼25*KO mice, their cerebella was significantly smaller than expected. The cerebellar folia from *miR-17∼92*cKO; *miR-106b∼25*KO and *miR-17∼92*cKO mice were misshapen with shallow fissures. Folia I–V and VI–VII were under developed when compared to cerebella of control mice (compare Fig. 3G and Fig. 3E with Fig. 3D, respectively, arrows). However, folia from the cerebella of *miR-106b∼25*KO animals appeared similar to those of controls (compare Fig. 3F with Fig. 3D). Scattered ectopic clusters of mature granule neurons were found on the surface of the molecular layer in *miR-17∼92*cKO; *miR-106b∼25*KO mice (Fig. 3G–I). The rest of the cerebellum cortical architecture appeared normal including a proper IGL, a monolayer of Purkinje cells with normal arborization, and Bergmann glia with normal fibers (negative data not shown). In spite of their small cerebella with foliation defects, *miR-17∼92*cKO; *miR-106b∼25*KO mice were asymptomatic with no evidence of gross neurological symptoms including motor coordination or balance defects measured on a Rotarod (negative data not shown).

**Fig. 4.**
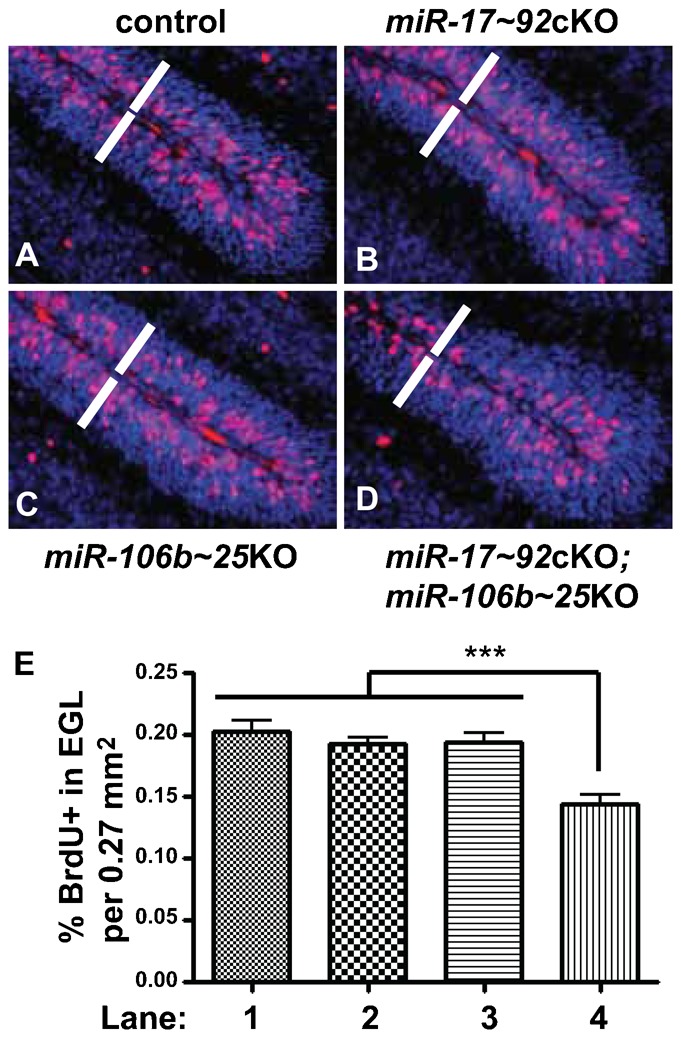
Reduced proliferation in cerebella of mice lacking the *miR-17∼92* and *miR-106b∼25* clusters. (A–D) Proliferation assessed by BrdU staining (red) on mid-sagittal sections through P7 cerebellum. Nuclei were counterstained with propidium iodide (blue). White bars indicate the EGL. (E) The percentage of BrdU+ cells in the EGL was quantified per 0.27 mm^2^. BrdU+ cells were counted in the EGL of lobule I and II from 2 sections per animal. Three mice for each genotype were analyzed. (***) p-values <0.001. (A and E, lane 1) control, (B and E, lane 2) *miR-17∼92*cKO, (C and E, lane 3) *miR-106b∼25*KO and (D and E, lane 4) *miR17∼92*cKO; *miR-106b∼25*KO.

**Fig. 5.**
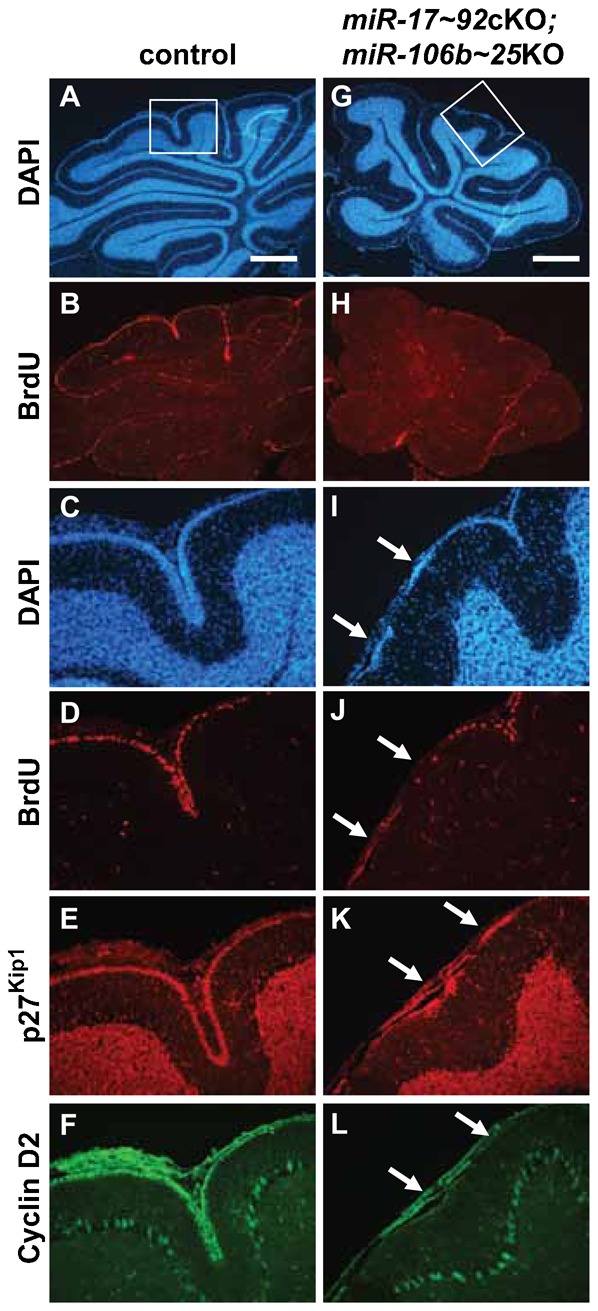
Premature termination of EGL proliferation in mice lacking the *miR-17∼92* and *miR-106b∼25* clusters. Mid-sagittal sections through the cerebellum of P14 control (A–F) and *miR-17∼92*cKO; *miR-106b∼25*KO (G–L) mice stained with DAPI (A,C,G,I), or antibodies raised against BrdU (B,D,H,J), p27^Kip1^ (E,K) and cyclin D2 (F,L). Enlarged views of boxed areas in panels A and G are presented in panels C–F and I–L, respectively. Arrows indicate 2 clusters of granule neurons out of cycle, on the surface of the cerebellum, in *miR-17∼92*cKO; *miR-106b∼25*KO mice. Scale bars: 500 µm.

Cerebellar development of *miR-17∼92*cKO; *miR-106b∼25*KO mice appeared normal during embryogenesis until birth, a time when GNPs respond to SHH to rapidly proliferate with maximal proliferation between P5 and P7. At P7, we found a significant reduction in BrdU incorporation in the EGL of cerebella from *miR-17∼92*cKO; *miR-106b∼25*KO mice compared to controls (Fig. 4D versus Fig. 4A and Fig. 4E) consistent with the requirement for massive proliferation of GNPs for proper foliation from birth until P7 (Lauder et al., 1974). The *miR-17∼92* cluster not only regulates proliferation but also apoptosis (Concepcion et al., 2012; Mogilyansky and Rigoutsos, 2013). We observed no significant differences in apoptosis by Terminal deoxynucleotidyl transferase dUTP nick end labeling staining between the cerebella of all 4 genotypes at P7 (negative data not shown). These results suggested that decreased proliferation of GNPs from cerebella of *miR-17∼92*cKO; *miR-106b∼25*KO mice might account for the diminished pool of GNPs during post-natal development. Consistent with this possibility, we observed premature exhaustion of proliferative GNPs at P14 in *miR-17∼92*cKO; *miR-106b∼25*KO mice. The EGL did not uniformly cover the surface of the cerebellum in contrast to that of control mice (compare Fig. 5G and Fig. 5I with Fig. 5A and Fig. 5C). The remaining cells in the EGL were mainly negative for BrdU and cyclin D2 but positive for p27^Kip1^, suggesting premature exit from the cell cycle and migration defects (Fig. 5H and Fig. 5J–L versus Fig. 5B and Fig. 5D–F).

### The *miR-17∼92* cluster is required for medulloblastoma formation

**Fig. 6.**
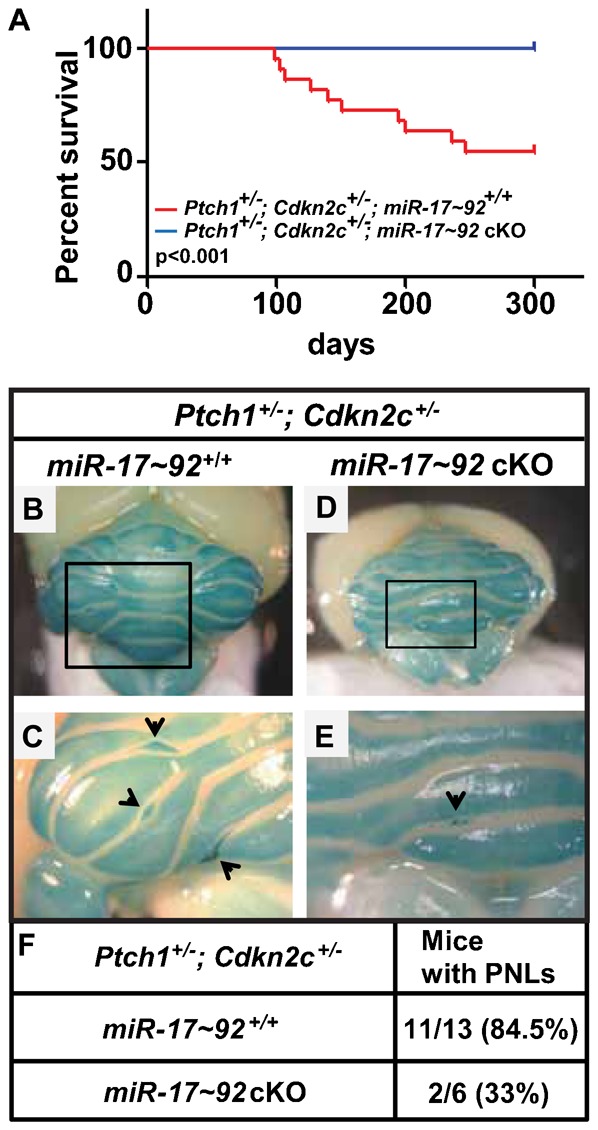
The *mir-17∼92* cluster is required for medulloblastoma formation. (A) Survival curves for *Ptch1^+/−^*; *Cdkn2c^+/−^* mice with different *miR-17∼92* and *Nestin-Cre* genotypes. *MiR-17*∼92cKO (blue line, n = 20) and *miR-17*∼92^+/+^ (red line, n = 22) mice. P-value <0.001. (B–E) In situ X-gal staining of cerebella from a 3 week old *Ptch1^+/−^*; *Cdkn2c^+/−^* mouse wild type (B,C) or null (D,E) for the *miR-17-92* cluster. Enlarged views of boxed areas in panels B and D are presented in panels C and E, respectively. (C) Arrows indicate 3 PNLs composed of densely packed cells. (E) Arrow indicates a region composed of scattered ectopic cells localized on the surface of the cerebellum. (F) Number of 3 week old mice revealing PNLs on the surface of their cerebella.

We previously reported that GNPs purified from *Ptch1^+/−^*; *Cdkn2c^−/−^* mice and overexpressing the *miR-17∼92* cluster induced SHH-MB after transplant in the cortex of naïve CD-1 *nu/nu* recipient mice (Uziel et al., 2009). However, unlike Mycn, enforced expression of the *miR-17∼92* cluster in GNPs purified from the cerebella of *Trp53^−/−^*; *Cdkn2c^−/−^* mice failed to induce SHH-MBs after orthotopic transplantation demonstrating that activation of the Patched signaling pathway was required for *miR-17∼92* induction of SHH-MBs (Uziel et al., 2009). To assess whether the *miR-17∼92* cluster was required for MB formation, we bred *miR-17∼92^floxed/+^*; *Nestin-Cre^+^* with *Ptch1^+/−^*; *Cdkn2c^−/−^* mice. While, as expected, 45.5% (10/22) of *miR-17∼92^+/+^*; *Ptch1^+/^*; *Cdkn2c^+/−^* mice succumbed to SHH-MBs, none (0/20) of the *miR-17∼92*cKO; *Ptch1^+/−^*, *Cdkn2c^+/−^* mice developed tumors over a period of 300 days (Fig. 6A).

**Fig. 7.**
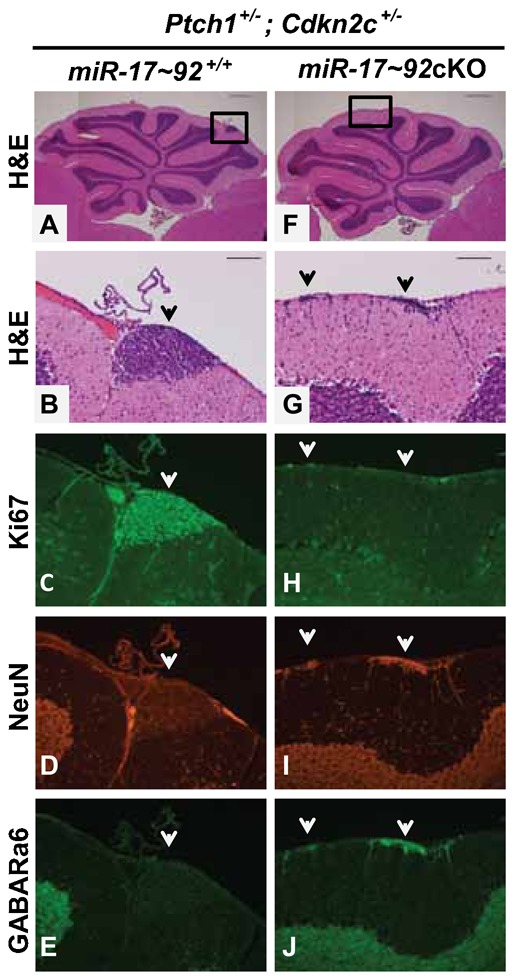
Foci of differentiated neurons at the cerebellar surface of 3 week old *miR-17∼92*cKO; *Ptch1^+/−^*; *Cdkn2c^+/−^* mice. Cerebella from 3 week old *Ptch1^+/−^*; *Cdkn2c^+/−^* mice wild type (A–E) or null (F–J) for the *miR-17∼92* cluster were stained with H&E (A,B,F,G), an antibody against Ki67 (C,H), NeuN (D,I), and GABA(A) receptor α6 subunit (GABARa6) (E,J). Enlarged views of boxed areas (A,F) are presented in panels B–E and G–J, respectively. PNL (arrow, B–E) is composed of densely packed undifferentiated and proliferating neurons. Foci of differentiated, non-proliferating neurons (arrows, G–J). Scale bars: 500 µm (A,F), 100 µm (B,G).

In wild-type mice, by 3 weeks after birth, all GNPs have exited the cell cycle and migrated from the surface of the cerebellum into the IGL. In contrast, in *Ptch1^+/−^* mice, several GNPs continue to divide and remain on the surface of the molecular layer (ML), to form clusters of densely packed cells called pre-neoplastic lesions (PNLs) (Goodrich et al., 1997; Kim et al., 2003; Oliver et al., 2005). In 6 weeks old mice, the majority of PNLs have regressed, while only few progress to MBs. To visualize PNLs, we stained the entire brains of 3 week old *miR-17∼92*cKO; *Ptch1^+/−^*, *Cdkn2c^+/−^* and *miR-17*∼92^+/+^; *Ptch1^+/−^*, *Cdkn2c^+/−^* mice with X-gal, since a portion of the wild-type allele of *Patched* is replaced by *LacZ* in the *Ptch1^+/−^* mice (Goodrich et al., 1997). We detected PNLs in 84.5% (11/13) *miR-17*∼92^+/+^; *Ptch1^+/−^*, *Cdkn2c^+/−^* mice (Fig. 6B,C,F). As expected, those PNLs were Ki67 positive but negative for NeuN and GABA (A) receptor α6 subunit (Fig. 7C–E, arrows). However, only 33% (2/6) of cerebella from *miR-17∼92*cKO; *Ptch1^+/−^*; *Cdkn2c^+/−^* mice showed PNLs (p = 0.0248) (Fig. 6F). Scattered foci of non-proliferating, differentiated neurons (negative for Ki67 but positive for NeuN and GABA (A) receptor α6 subunit) were observed on the surface of the cerebellar ML of *miR-17∼92*cKO; *Ptch1^+/−^*; *Cdkn2c^+/−^* mice (Fig. 6D,E, Fig. 7H–J, arrows). These results suggest that the *miR-17∼92* cluster is required for SHH-induced PNL formation and for SHH-MB development.

**Fig. 8.**
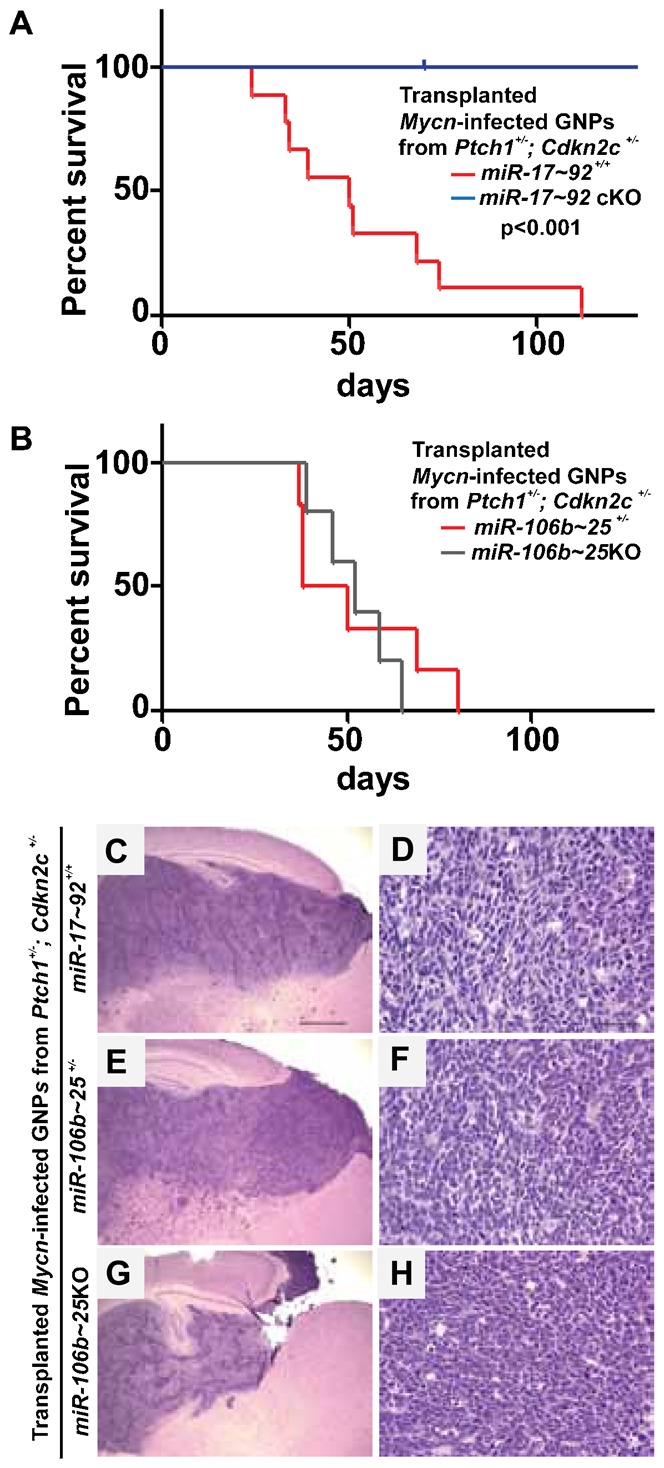
Enforced *Mycn* expression in GNPs from *Ptch1^+/−^*; *Cdkn2c^+/−^* but not from *miR-17∼92*cKO; *Ptch1^+/−^*; *Cdkn2c^+/−^* mice induces SHH-MBs. (A) Survival curves for naïve CD-1 *nu/nu* mice transplanted with GNPs purified from the cerebella of P7 *miR-17∼92*cKO; *Ptch1^+/−^*; *Cdkn2c^+/−^* (blue line, n = 12) or from *miR-17∼92*^+/+^; *Ptch1^+/−^*; *Cdkn2c^+/−^* (red line, n = 9) mice and infected with retroviruses expressing *Mycn*. P-value <0.001. (B) Survival curves for naïve CD-1 *nu/nu* mice transplanted with GNPs purified from the cerebella of P7 *miR-106b∼25*KO; *Ptch1^+/−^*; *Cdkn2c^+/−^* (grey line, n = 5) or from *miR-106b∼25*^+/−^; *Ptch1^+/−^*; *Cdkn2c^+/−^* (red line, n = 6) mice and infected with retroviruses expressing *Mycn*. (C–H) H&E staining of medulloblastoma after cortical implants of GNPs purified from the cerebella of P7 *miR-17∼92^+/+^*; *Ptch1^+/−^*; *Cdkn2c^+/−^* (C,D) or *miR-106b∼25*^+/−^; *Ptch1^+/−^*; *Cdkn2c^+/−^* (E,F) or *miR-106b∼25*KO; *Ptch1^+/−^*; *Cdkn2c^+/−^* mice (G,H), infected with retroviruses expressing *Mycn*. Scale bars: 1 mm (C,E,G) and 50 µm (D,F,H).

Mycn is a direct target of SHH signaling and is required for SHH-MB formation (Kenney et al., 2003; Hatton et al., 2006). Using an orthotopic transplantation approach, we previously showed that enforced expression of *Mycn* in GNPs purified from P7 cerebella of *Ptch1^+/−^*, *Cdkn2c^−/−^* mice induces SHH-MBs (Zindy et al., 2007; Kawauchi et al., 2012). GNPs purified from the cerebella of P7 *miR-17∼92*cKO; *Ptch1^+/−^*; *Cdkn2c^+/−^* and *miR-17∼*92^+/+^; *Ptch1^+/−^*; *Cdkn2c^+/−^* mice were infected with retroviruses encoding *Mycn* and the red fluorescence protein (RFP), and stereotactically implanted 2 days later into the cortices of naïve recipient animals. Mycn expression did not increase apoptosis or cell cycle arrest in infected GNPs after three days in culture (negative data not shown). As expected, 100% (9/9) of the animals transplanted with GNPs purified from the cerebella of P7 *miR-17∼*92^+/+^; *Ptch1^+/−^*; *Cdkn2c^+/−^* mice and overexpressing *Mycn* developed SHH-MBs (Fig. 8A) (Zindy et al., 2007; Kawauchi et al., 2012). In contrast, none of the 12 mice transplanted with GNPs purified from the cerebella of P7 *miR-17∼92*cKO; *Ptch1^+/−^*; *Cdkn2c^+/−^* mice and expressing *Mycn* developed medulloblastoma, 180 days post-implantation. However, 5/5 mice transplanted with GNPs purified from the cerebella of P7 *miR-106b∼25*KO; *Ptch1^+/−^*; *Cdkn2c^+/−^* mice in which we enforced *Mycn* expression developed MBs (Fig. 8B). Pathological analysis of the tumors of all genotypes confirmed that they were MBs of the SHH subtype with classic morphology of round cells (Fig. 8C–H), as previously described (Zindy et al., 2007; Kawauchi et al., 2012).

These results point to an absolute requirement for the *miR-17∼92*, but not for the *miR-106b∼25*, cluster in SHH-MB initiation.

### Targets of the *miR-17∼92* family cluster

**Fig. 9.**
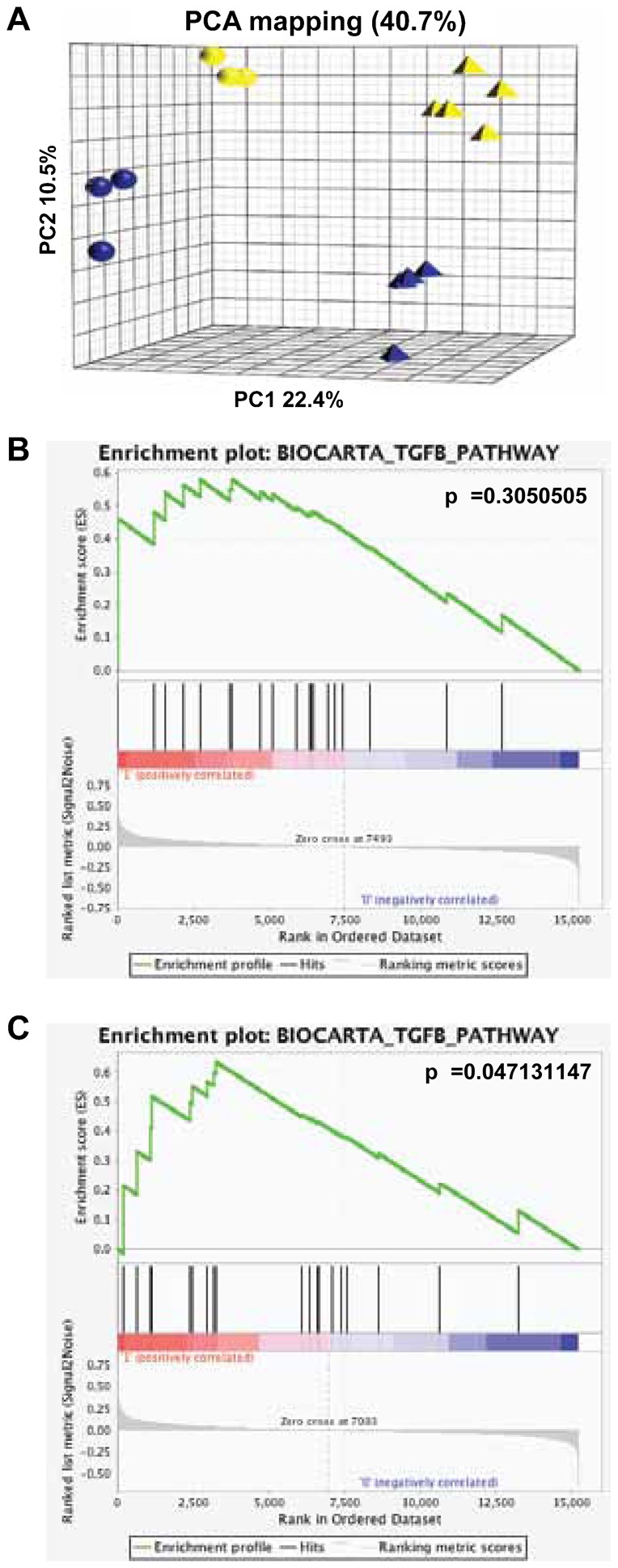
The *miR-17∼92* cluster suppresses TGF-β signaling. (A) Principal component analysis (PCA) of cerebella from P4 (blue spheres, n = 3) and P7 (blue pyramids, n = 4) control mice, and from P4 (yellow spheres, n = 3) and P7 (yellow pyramids, n = 5) *miR-17∼92*cKO; *miR-106b∼25*KO mice. (B,C) Gene set enrichment analysis plot for Biocarta TGF-β responsive genes set between cerebella from P7 *miR-17∼92*cKO; *miR-106b∼25*KO and P7 control mice (B; p-value = 0.3050) and between cerebella from P4 and P7 *miR-17∼92*cKO; *miR-106b∼25*KO and P4 and P7 control mice (C; p-value = 0.04713).

We recently reported that the *miR-17∼92* cluster down-regulates bone morphogenic protein (Bmp) receptor type 2 (Bmpr2), the receptor for Bmp-2, -4 and -7 and that higher levels of Bmpr2 are detected in cerebella of P7 *miR-17∼92*cKO; *miR-106b∼25*KO mice compared to those of controls (Murphy et al., 2013). To identify potential targets of the *miR-17∼92* cluster family, we compared the gene expression profile of cerebella from control and *miR-17∼92*cKO; *miR-106b∼25*KO mice at P4, and P7, times at which difference in cerebellar size was detectable. Principal component analysis revealed that cerebella from control mice clustered together but independently from those of *miR-17∼92*cKO; *miR-106b∼25*KO mice for each time point (Fig. 9A). To gain insights into the pathways affected by the deletion of *miR-17∼92* and *miR-106b∼25* clusters we performed GSEA. Using the Biocarta gene sets, we found that the Transforming Growth Factor (TGF)-β responsive signaling pathway was enriched in cerebella from P7 *miR-17∼92*cKO; *miR-106b∼25*KO mice relatively to controls (Fig. 9B). Although these results were not statistically significant, the TGF-β responsive gene set was significantly enriched when we combined P4 and P7 cerebella (Fig. 9C) suggesting that this pathway was responsible in part for the reduced proliferation, as previously reported (Aref et al., 2013).

## DISCUSSION

Here we analyzed the role of the *miR-17∼92* cluster family during cerebellar development by conditional deletion the *miR-17∼92* cluster alone or together with the *miR-106b∼25* cluster (*miR-17∼92*cKO; *miR-106b∼25*KO) in neural progenitors. While lack of the *miR-106b∼25* cluster had no obvious phenotype, loss of the *miR-17∼92* cluster induced a reduction in cerebellar size and foliation. Loss of both clusters induced a more severe phenotype. Deletion of the *miR-17∼92* cluster was sufficient to completely abolish tumor development, pointing to the absolute requirement for the *miR-17∼92* cluster, and to the lack of compensation by the *miR-106b∼25* cluster, for MB development in collaboration with constitutively activated SHH signaling.

### The *miR-17∼92* and *miR106b∼25* clusters control the number of GNPs during post-natal cerebellar development

We previously reported that microRNAs from the *miR-106a∼363* cluster are not expressed in wild-type cerebella (Uziel et al., 2009). We found no significant up-regulation of microRNAs encoded by this cluster in cerebella lacking both *miR-17∼92* and *miR-106b∼25* clusters (data not shown). This suggested that the *miR-106a∼363* cluster is unlikely to contribute to the phenotype seen in *miR-17∼92*cKO; *miR-106b∼25*KO mice.

Co-deletion of the two clusters *miR-17∼92* and *miR-106b∼25* led to the exhaustion of progenitor neurons in the cerebellum resulting in reduction in cerebellar size and the number of folia. The *miR-17∼92* cluster controls the number of oligodendroglial cells (Budde et al., 2010), renal tubular cells (Patel et al., 2013), neural stem cells (Bian et al., 2013) and cardiomyocytes (Chen et al., 2013). However, in contrast, the *miR-17∼92* cluster alone or in combination with *miR-106b∼25* is dispensable during retinal development (Conkrite et al., 2011; Nittner et al., 2012), mammary development (Feuermann et al., 2012) and Langerhans cells (Zhou et al., 2014). Thus, the requirement for microRNAs encoded by the *miR-17∼92* cluster is cell context specific.

The *miR-17∼92* and *miR-106b∼25* clusters are direct targets of Myc and Mycn (O'Donnell et al., 2005; Northcott et al., 2009; de Pontual et al., 2011). In the central nervous system, the loss of *Mycn* in Nestin-positive cells induces a reduced size and misformed cerebellum (Knoepfler et al., 2002). However, in these mice, the proliferation of the residual GNPs is driven by the up-regulation of *Myc* that normally is not expressed in these neuronal progenitors (Zindy et al., 2006). Expansion of GNPs in postnatal cerebella of mice deficient for both *Mycn* and *Myc* is severely reduced leading to a cerebellum lacking all folia (Wey et al., 2010). The fact that the loss of the *miR-17∼92* and *miR-106b∼25* clusters attenuated proliferation of GNPs but still caused folia formation suggests that both clusters are required but not sufficient to induce the cerebellar phenotype of *Mycn*; *Myc* double-null mice.

### The *miR-17∼92* cluster is absolutely required for SHH-MB development

*MiRs* encoded by the *miR-17∼92* cluster are overexpressed in both mouse and human tumors (Uziel et al., 2009; Northcott et al., 2009). We previously found that enforced expression of the *miR-17∼92* cluster collaborates with SHH signaling to induce SHH-MB (Uziel et al., 2009). Strikingly, deletion of the *miR-17∼92* cluster in Nestin-positive cells from *Ptch1^+/−^*; *Cdkn2c^+/−^* mice completely abolished SHH-MB development. This was associated with a significant decrease in the number of 3 week old *Ptch1^+/−^*; *Cdkn2c^+/−^* mice lacking the *miR-17∼92* cluster that exhibit PNLs on the surface of their cerebella. Therefore, the *miR-17∼92* cluster plays a critical role in GNPs during SHH-MB initiation as was shown in retinoblastoma (Nittner et al., 2012).

**Fig. 10.**
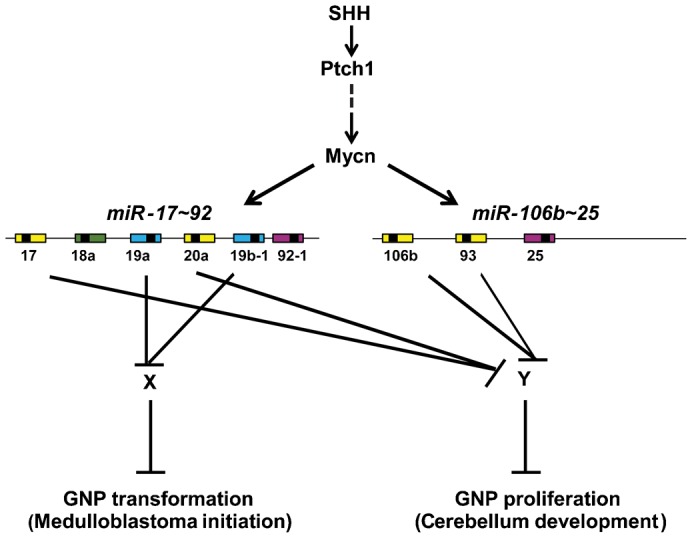
Schematic representation of the *miR-17∼92* cluster family and its regulation by SHH signaling. SHH signaling induces the transcription of *Mycn*, which, in turn, induces the expression of the *miR-17∼92* and *miR-106b∼25* clusters that encode 6 and 3 microRNAs, respectively. MicroRNAs sharing the same seed sequence are represented by boxes of the same color. X and Y represent putative targets. Loss of the *miR-17∼92* but not the *miR-106b∼25* cluster inhibits *Ptch1^+/−^*-induced SHH-MBs. Loss of both clusters reduces cerebellar size.

Our data also showed that the *miR-106b∼25* did not compensate for the loss of the *miR-17∼92* cluster in tumor initiation. Analysis of the microRNAs expressed by the two clusters revealed that *miR-19a* and *miR-19b-1* that share the same seed sequence are encoded only by the *miR-17∼92* but not the *miR-106b∼25* cluster (Fig. 10). This suggests that these two microRNAs might be sufficient for tumor development and that they might recapitulate the oncogenic function of the *miR-17∼92* cluster, as reported previously for the development of Eμ-Myc B cell lymphoma (Mu et al., 2009; Olive et al., 2009). Treatment of SHH-MB with tiny LNAs directed against *miR-17/20a/106b/93* and *miR-19a/b-1* from the *miR-17∼92* cluster family inhibits proliferation of SHH-MB *in vitro* and *in vivo* (Murphy et al., 2013). Therefore the *miR-19a/b-1* might not be the only microRNA required for MB progression, although this will need further evaluation.

Given the absolute requirement of the *miR-17∼92* cluster for *Ptch1^+/−^* induced SHH-MB formation, the identification of bone fide *miR-17∼92* targets is clearly warranted. Analysis of previously published targets (Brock et al., 2009; Concepcion et al., 2012; Mogilyansky and Rigoutsos, 2013; Sun et al., 2013) revealed that Bmpr2, but not PTEN and p21^Cip1^, is regulated by the *miR-17∼92* cluster (Murphy et al., 2013). We found that Bmpr2 was upregulated in the cerebella of *miR-17∼92*cKO; *miR-106b∼25*KO mice, which might be responsible, in part, for the cerebellar anomalies. Constitutive activation of Bmpr1a in cerebella induced a simplified foliation pattern (Ming et al., 2002). These data are consistent with ours and others findings showing that BMP signaling antagonizes the SHH pathway (Rios et al., 2004) and that genes in the BMP pathway are downregulated in SHH-MB (Zhao et al., 2008). It has been previously reported that the *miR-17∼92* cluster down-regulates multiple components of the TGF-β pathway (Tagawa et al., 2007; Petrocca et al., 2008; Dews et al., 2010; Mestdagh et al., 2010; Concepion et al., 2012; Li et al., 2012; Mogilyansky and Rigoutsos, 2013). In agreement with these reports, we found, by GSEA analysis, that the TGF-β pathway was upregulated in the cerebella of *miR-17∼92*cKO; *miR-106b∼25*KO mice. TGF-β pathway is associated with SHH-MB pathogenesis with high levels connoting a good prognosis (Aref et al., 2013). Our results are in agreement with a study suggesting that *miR-17∼92* cluster downregulates two different signaling pathways, Bmp and TGF-β pathways (Brock et al., 2009). Further analysis using Clip-Seq approaches will be required to identify the bona fide *miR-17∼92* targets in mouse and human MBs (Darnell, 2010).

In summary, while the *miR-17∼92* and *miR-106b∼25* clusters are essential for cerebellum development and homeostasis, the *miR-17∼92*, but not the *miR-106b∼25*, cluster is required for tumor initiation in *Ptch1^+/−^* mice.

## Supplementary Material

Supplementary Material

## References

[b1] Aref D. Moffatt C. J. Agnihotri S. Ramaswamy V. Dubuc A. M. Northcott P. A. Taylor M. D. Perry A. Olson J. M. Eberhart C. G. (2013). Canonical TGF-β pathway activity is a predictor of SHH-driven medulloblastoma survival and delineates putative precursors in cerebellar development. Brain Pathol. 23, 178–191 10.1111/j.1750-3639.2012.00631.x 22966790 PMC8029114

[b2] Bian S. Hong J. Li Q. Schebelle L. Pollock A. Knauss J. L. Garg V. Sun T. (2013). MicroRNA cluster miR-17-92 regulates neural stem cell expansion and transition to intermediate progenitors in the developing mouse neocortex. Cell Rep. 3, 1398–1406 10.1016/j.celrep.2013.03.037 23623502 PMC3762321

[b3] Brock M. Trenkmann M. Gay R. E. Michel B. A. Gay S. Fischler M. Ulrich S. Speich R. Huber L. C. (2009). Interleukin-6 modulates the expression of the bone morphogenic protein receptor type II through a novel STAT3-microRNA cluster 17/92 pathway. Circ. Res. 104, 1184–1191 10.1161/CIRCRESAHA.109.197491 19390056

[b4] Budde H. Schmitt S. Fitzner D. Opitz L. Salinas-Riester G. Simons M. (2010). Control of oligodendroglial cell number by the miR-17-92 cluster. Development 137, 2127–2132 10.1242/dev.050633 20504959

[b5] Carmell M. A. Hannon G. J. (2004). RNase III enzymes and the initiation of gene silencing. Nat. Struct. Mol. Biol. 11, 214–218 10.1038/nsmb729 14983173

[b6] Chen J. Huang Z. P. Seok H. Y. Ding J. Kataoka M. Zhang Z. Hu X. Wang G. Lin Z. Wang S. (2013). mir-17-92 cluster is required for and sufficient to induce cardiomyocyte proliferation in postnatal and adult hearts. Circ. Res. 112, 1557–1566 10.1161/CIRCRESAHA.112.300658 23575307 PMC3756475

[b7] Concepcion C. P. Bonetti C. Ventura A. (2012). The microRNA-17-92 family of microRNA clusters in development and disease. Cancer J. 18, 262–267 10.1097/PPO.0b013e318258b60a 22647363 PMC3592780

[b8] Conkrite K. Sundby M. Mukai S. Thomson J. M. Mu D. Hammond S. M. MacPherson D. (2011). *miR-17∼92* cooperates with RB pathway mutations to promote retinoblastoma. Genes Dev. 25, 1734–1745 10.1101/gad.17027411 21816922 PMC3165937

[b9] Darnell R. B. (2010). HITS-CLIP: panoramic views of protein-RNA regulation in living cells. Wiley Interdiscip Rev RNA 1, 266–286 10.1002/wrna.31 21935890 PMC3222227

[b10] de Pontual L. Yao E. Callier P. Faivre L. Drouin V. Cariou S. Van Haeringen A. Geneviève D. Goldenberg A. Oufadem M. (2011). Germline deletion of the miR-17∼92 cluster causes skeletal and growth defects in humans. Nat. Genet. 43, 1026–1030 10.1038/ng.915 21892160 PMC3184212

[b11] Dews M. Fox J. L. Hultine S. Sundaram P. Wang W. Liu Y. Y. Furth E. Enders G. H. El-Deiry W. Schelter J. M. (2010). The myc-miR-17∼92 axis blunts TGFbeta signaling and production of multiple TGFbeta-dependent antiangiogenic factors. Cancer Res. 70, 8233–8246 10.1158/0008-5472.CAN-10-2412 20940405 PMC3007123

[b12] Feuermann Y. Robinson G. W. Zhu B. M. Kang K. Raviv N. Yamaji D. Hennighausen L. (2012). The miR-17/92 cluster is targeted by STAT5 but dispensable for mammary development. Genesis 50, 665–671 10.1002/dvg.22023 22389215 PMC3560854

[b13] Goodrich L. V. Milenković L. Higgins K. M. Scott M. P. (1997). Altered neural cell fates and medulloblastoma in mouse patched mutants. Science 277, 1109–1113 10.1126/science.277.5329.1109 9262482

[b14] Hatten M. E. Roussel M. F. (2011). Development and cancer of the cerebellum. Trends Neurosci. 34, 134–142 10.1016/j.tins.2011.01.002 21315459 PMC3051031

[b15] Hatton B. A. Knoepfler P. S. Kenney A. M. Rowitch D. H. de Alborán I. M. Olson J. M. Eisenman R. N. (2006). N-myc is an essential downstream effector of Shh signaling during both normal and neoplastic cerebellar growth. Cancer Res. 66, 8655–8661 10.1158/0008-5472.CAN-06-1621 16951180

[b16] Kawauchi D. Robinson G. Uziel T. Gibson P. Rehg J. Gao C. Finkelstein D. Qu C. Pounds S. Ellison D. W. (2012). A mouse model of the most aggressive subgroup of human medulloblastoma. Cancer Cell 21, 168–180 10.1016/j.ccr.2011.12.023 22340591 PMC3285412

[b17] Kenney A. M. Cole M. D. Rowitch D. H. (2003). Nmyc upregulation by sonic hedgehog signaling promotes proliferation in developing cerebellar granule neuron precursors. Development 130, 15–28 10.1242/dev.00182 12441288

[b18] Kim V. N. (2005). MicroRNA biogenesis: coordinated cropping and dicing. Nat. Rev. Mol. Cell Biol. 6, 376–385 10.1038/nrm1644 15852042

[b19] Kim J. Y. Nelson A. L. Algon S. A. Graves O. Sturla L. M. Goumnerova L. C. Rowitch D. H. Segal R. A. Pomeroy S. L. (2003). Medulloblastoma tumorigenesis diverges from cerebellar granule cell differentiation in patched heterozygous mice. Dev. Biol. 263, 50–66 10.1016/S0012-1606(03)00434-2 14568546

[b20] Knoepfler P. S. Cheng P. F. Eisenman R. N. (2002). N-myc is essential during neurogenesis for the rapid expansion of progenitor cell populations and the inhibition of neuronal differentiation. Genes Dev. 16, 2699–2712 10.1101/gad.1021202 12381668 PMC187459

[b21] Lauder J. M. Altman J. Krebs H. (1974). Some mechanisms of cerebellar foliation: effects of early hypo- and hyperthyroidism. Brain Res. 76, 33–40 10.1016/0006-8993(74)90511-3 4844095

[b22] Li L. Shi J. Y. Zhu G. Q. Shi B. (2012). MiR-17-92 cluster regulates cell proliferation and collagen synthesis by targeting TGFB pathway in mouse palatal mesenchymal cells. J. Cell. Biochem. 113, 1235–1244 10.1002/jcb.23457 22095742

[b23] Mestdagh P. Boström A. K. Impens F. Fredlund E. Van Peer G. De Antonellis P. von Stedingk K. Ghesquière B. Schulte S. Dews M. (2010). The miR-17-92 microRNA cluster regulates multiple components of the TGF-β pathway in neuroblastoma. Mol. Cell 40, 762–773 10.1016/j.molcel.2010.11.038 21145484 PMC3032380

[b24] Ming J. E. Elkan M. Tang K. Golden J. A. (2002). Type I bone morphogenetic protein receptors are expressed on cerebellar granular neurons and a constitutively active form of the type IA receptor induces cerebellar abnormalities. Neuroscience 114, 849–857 10.1016/S0306-4522(02)00348-2 12379241

[b25] Mogilyansky E. Rigoutsos I. (2013). The miR-17/92 cluster: a comprehensive update on its genomics, genetics, functions and increasingly important and numerous roles in health and disease. Cell Death Differ. 20, 1603–1614 10.1038/cdd.2013.125 24212931 PMC3824591

[b26] Mu P. Han Y. C. Betel D. Yao E. Squatrito M. Ogrodowski P. de Stanchina E. D'Andrea A. Sander C. Ventura A. (2009). Genetic dissection of the miR-17∼92 cluster of microRNAs in Myc-induced B-cell lymphomas. Genes Dev. 23, 2806–2811 10.1101/gad.1872909 20008931 PMC2800095

[b27] Murphy B. L. Obad S. Bihannic L. Ayrault O. Zindy F. Kauppinen S. Roussel M. F. (2013). Silencing of the miR-17∼92 cluster family inhibits medulloblastoma progression. Cancer Res. 73, 7068–7078 10.1158/0008-5472.CAN-13-0927 24145352 PMC3857104

[b28] Nittner D. Lambertz I. Clermont F. Mestdagh P. Köhler C. Nielsen S. J. Jochemsen A. Speleman F. Vandesompele J. Dyer M. A. (2012). Synthetic lethality between Rb, p53 and Dicer or miR-17-92 in retinal progenitors suppresses retinoblastoma formation. Nat. Cell Biol. 14, 958–965 10.1038/ncb2556 22864477

[b29] Northcott P. A. Fernandez-L A. Hagan J. P. Ellison D. W. Grajkowska W. Gillespie Y. Grundy R. Van Meter T. Rutka J. T. Croce C. M. (2009). The miR-17/92 polycistron is up-regulated in sonic hedgehog-driven medulloblastomas and induced by N-myc in sonic hedgehog-treated cerebellar neural precursors. Cancer Res. 69, 3249–3255 10.1158/0008-5472.CAN-08-4710 19351822 PMC2836891

[b30] O'Donnell K. A. Wentzel E. A. Zeller K. I. Dang C. V. Mendell J. T. (2005). c-Myc-regulated microRNAs modulate E2F1 expression. Nature 435, 839–843 10.1038/nature03677 15944709

[b31] Olive V. Bennett M. J. Walker J. C. Ma C. Jiang I. Cordon-Cardo C. Li Q. J. Lowe S. W. Hannon G. J. He L. (2009). miR-19 is a key oncogenic component of mir-17-92. Genes Dev. 23, 2839–2849 10.1101/gad.1861409 20008935 PMC2800084

[b32] Oliver T. G. Read T. A. Kessler J. D. Mehmeti A. Wells J. F. Huynh T. T. Lin S. M. Wechsler-Reya R. J. (2005). Loss of patched and disruption of granule cell development in a pre-neoplastic stage of medulloblastoma. Development 132, 2425–2439 10.1242/dev.01793 15843415

[b33] Patel V. Williams D. Hajarnis S. Hunter R. Pontoglio M. Somlo S. Igarashi P. (2013). miR-17∼92 miRNA cluster promotes kidney cyst growth in polycystic kidney disease. Proc. Natl. Acad. Sci. USA 110, 10765–10770 10.1073/pnas.1301693110 23759744 PMC3696812

[b34] Petrocca F. Vecchione A. Croce C. M. (2008). Emerging role of miR-106b-25/miR-17-92 clusters in the control of transforming growth factor beta signaling. Cancer Res. 68, 8191–8194 10.1158/0008-5472.CAN-08-1768 18922889

[b35] Rios I. Alvarez-Rodríguez R. Martí E. Pons S. (2004). Bmp2 antagonizes sonic hedgehog-mediated proliferation of cerebellar granule neurones through Smad5 signalling. Development 131, 3159–3168 10.1242/dev.01188 15197161

[b36] Sun Q. Mao S. Li H. Zen K. Zhang C. Y. Li L. (2013). Role of miR-17 family in the negative feedback loop of bone morphogenetic protein signaling in neuron. PLoS ONE 8, e83067 10.1371/journal.pone.0083067 24349434 PMC3859655

[b37] Tagawa H. Karube K. Tsuzuki S. Ohshima K. Seto M. (2007). Synergistic action of the microRNA-17 polycistron and Myc in aggressive cancer development. Cancer Sci. 98, 1482–1490 10.1111/j.1349-7006.2007.00531.x 17608773 PMC11159226

[b38] Taylor M. D. Northcott P. A. Korshunov A. Remke M. Cho Y. J. Clifford S. C. Eberhart C. G. Parsons D. W. Rutkowski S. Gajjar A. (2012). Molecular subgroups of medulloblastoma: the current consensus. Acta Neuropathol. 123, 465–472 10.1007/s00401-011-0922-z 22134537 PMC3306779

[b39] Tronche F. Kellendonk C. Kretz O. Gass P. Anlag K. Orban P. C. Bock R. Klein R. Schütz G. (1999). Disruption of the glucocorticoid receptor gene in the nervous system results in reduced anxiety. Nat. Genet. 23, 99–103 10.1038/12703 10471508

[b40] Uziel T. Zindy F. Xie S. Lee Y. Forget A. Magdaleno S. Rehg J. E. Calabrese C. Solecki D. Eberhart C. G. (2005). The tumor suppressors Ink4c and p53 collaborate independently with Patched to suppress medulloblastoma formation. Genes Dev. 19, 2656–2667 10.1101/gad.1368605 16260494 PMC1283959

[b41] Uziel T. Karginov F. V. Xie S. Parker J. S. Wang Y. D. Gajjar A. He L. Ellison D. Gilbertson R. J. Hannon G. (2009). The miR-17∼92 cluster collaborates with the Sonic Hedgehog pathway in medulloblastoma. Proc. Natl. Acad. Sci. USA 106, 2812–2817 10.1073/pnas.0809579106 19196975 PMC2636735

[b42] Ventura A. Young A. G. Winslow M. M. Lintault L. Meissner A. Erkeland S. J. Newman J. Bronson R. T. Crowley D. Stone J. R. (2008). Targeted deletion reveals essential and overlapping functions of the miR-17 through 92 family of miRNA clusters. Cell 132, 875–886 10.1016/j.cell.2008.02.019 18329372 PMC2323338

[b43] Wey A. Martinez Cerdeno V. Pleasure D. Knoepfler P. S. (2010). c- and N-myc regulate neural precursor cell fate, cell cycle, and metabolism to direct cerebellar development. Cerebellum 9, 537–547 10.1007/s12311-010-0190-9 20658325 PMC2996535

[b44] Zhao H. Ayrault O. Zindy F. Kim J. H. Roussel M. F. (2008). Post-transcriptional down-regulation of Atoh1/Math1 by bone morphogenic proteins suppresses medulloblastoma development. Genes Dev. 22, 722–727 10.1101/gad.1636408 18347090 PMC2275424

[b45] Zhou L. Qi R. Q. Liu M. Xu Y. P. Li G. Weiland M. Kaplan D. H. Mi Q. S. (2014). microRNA miR-17-92 cluster is highly expressed in epidermal Langerhans cells but not required for its development. Genes Immun. 15, 57–61 10.1038/gene.2013.61 24285176

[b46] Zindy F. Knoepfler P. S. Xie S. Sherr C. J. Eisenman R. N. Roussel M. F. (2006). N-Myc and the cyclin-dependent kinase inhibitors p18Ink4c and p27Kip1 coordinately regulate cerebellar development. Proc. Natl. Acad. Sci. USA 103, 11579–11583 10.1073/pnas.0604727103 16864777 PMC1518798

[b47] Zindy F. Uziel T. Ayrault O. Calabrese C. Valentine M. Rehg J. E. Gilbertson R. J. Sherr C. J. Roussel M. F. (2007). Genetic alterations in mouse medulloblastomas and generation of tumors de novo from primary cerebellar granule neuron precursors. Cancer Res. 67, 2676–2684 10.1158/0008-5472.CAN-06-3418 17363588

