# Supplementary material for: Role of the miR-17∼92 cluster family in cerebellar and medulloblastoma development
Source: Biol Open. 2014 Jun 13;3(7):597–605. doi: 10.1242/bio.20146734 (PMC4154296; doi:10.1242/bio.20146734)
Supplement: Supplementary Material [file supp_3_7_597__index.html]

Role of the miR-17∼92 cluster family in cerebellar and medulloblastoma development — Supplementary Material 

# Role of the *miR-17∼92* cluster family in cerebellar and medulloblastoma development

## bio.20146734 Supplementary Material

**Files in this Data Supplement:**

- Supplementary Material - Frederique Zindy et al. doi: 10.1242/bio.20146734
