# Supplementary material for: Role of the miR-17∼92 cluster family in cerebellar and medulloblastoma development
Source: Biol Open. 2014 Jun 13;3(7):597–605. doi: 10.1242/bio.20146734 (PMC4154296; doi:10.1242/bio.20146734)
Supplement: Supplementary Material [file supp_bio.20146734_bio.20146734-s1.pdf]

Biology Open (2014) 000, 1–9 doi:10.1242/bio.20146734

Supplementary Material

Frederique Zindy et al. doi: 10.1242/bio.20146734

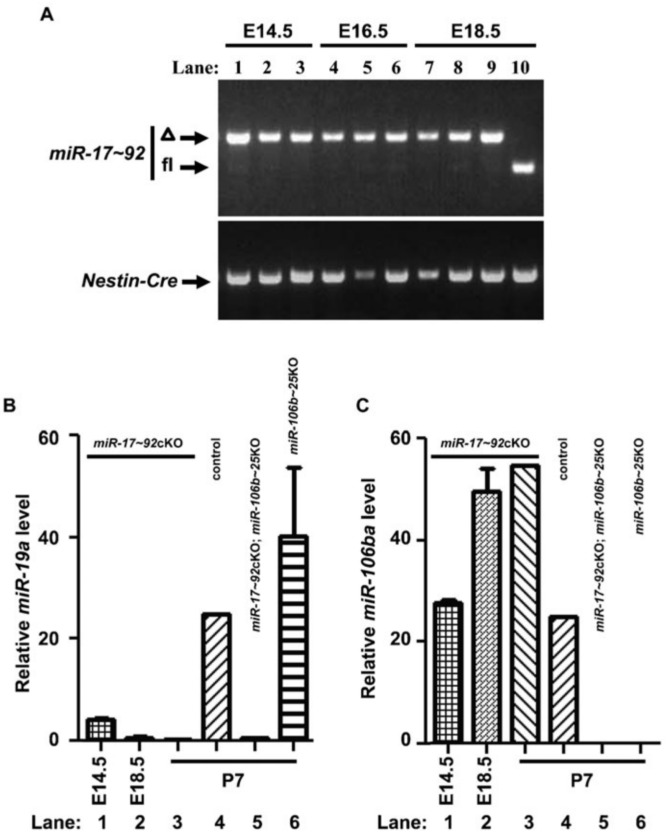

Fig. S1. Relative expression of *miR-19a* and *miR-106b* in *miR-17~92*cKO; *miR-106b~25*KO mice. (A) PCR for *miR-17~92* and *Nestin-Cre* was performed on DNA extracted from total cerebellum (lanes 1–9) or tail (lane 10) from *miR-17~92*cKO mice (lanes 1–10) at E14.5 (lanes 1–3), E16.5 (lanes 4–6) and E18.5 (lanes 7–10). (fl) floxed and (Δ) deleted *mir-17~92* alleles. Relative levels of mature microRNA *miR-19a* (B) and *miR-106b* (C) were determined by Q-RT-PCR on total RNAs extracted from total cerebella (lanes 1, 2, 5, 6) or purified granule cell precursors (lanes 3, 4). Lanes 1, 2 and 3 correspond to *mir-17~92*cKO at E14.5, E18.5 and P7, respectively, lane 4 to *miR-17~92* control at P7, lane 5 to *mir-17~92*cKO; *miR-106b~25*KO at P7 and lane 6 to *mir-106b~25*KO at P7.
